# Supplementary material for: GmTCP40 Promotes Soybean Flowering under Long-Day Conditions by Binding to the GmAP1a Promoter and Upregulating Its Expression
Source: Biomolecules. 2024 Apr 10;14(4):465. doi: 10.3390/biom14040465 (PMC11047976; doi:10.3390/biom14040465)
Supplement: Supplementary file 1 [file biomolecules-14-00465-s001.zip › biomolecules-2914325-supplementary.pdf]

Supplementary Materials

Supplementary Methods

Supplementary Figure S1

Supplementary Figure S2

Supplementary Figure S3

Supplementary Figure S4

Supplementary Table S1

Supplementary Table S2

Supplementary Table S3

Supplementary Table S4

Supplementary Table S5

Supplementary Methods

Protocol for transformation of the recombinant vector into *Agrobacterium tumefaciens* EHA101/GV3101/EHA105/EHA105 (pSoup):

Thaw the chemically competent cells on ice.

Gently mix the thawed cells with the recombinant vector using a pipette.

Sequentially incubate the mixture on ice for 10 min, in liquid nitrogen for 5 min, in a 37°C water bath for 30 s, and on ice for another 10 min.

Add 700 µL of antibiotic-free Luria-Bertani (LB) liquid medium to the mixture and shake the culture at 28°C for 2–3 h.

Centrifuge the culture at 6000 rpm for 1 min to pellet the bacteria.

Retain about 100 µL of the supernatant and use it to gently resuspend the bacterial pellet.

Spread the resuspended bacterial cells onto LB agar plates containing the appropriate antibiotic.

Invert the plates and incubate at 28°C for 2–3 days.

Protocol for transformation into the yeast strain EGY48:

Thaw 100 µL of EGY48 competent cells on ice.

Add pre-chilled plasmids to the cells, followed by 10 µL Carrier DNA. Heat the mixture at 95–100°C for 5 min, then place in an ice bath.

Repeat the heating and chilling steps once then add 500 µL of PEG/LiAc and gently mix by pipetting.

Incubate the mixture in a water bath at 30°C for 30 min (inverting the tube 6–8 times every 15 min).

Place the tube in a 42 °C water bath for 15 min (inverting 6–8 times every 7.5 min).

Centrifuge at 5000 rpm for 40 s and discard the supernatant.

Resuspend the cells in 400 µL of ddH_2O and centrifuge for 30 s before discarding the supernatant.

Resuspend the cells in 50 µL of ddH_2O, spread onto SD/-Trp/-Ura plates, and incubate at 30°C for 3–5 days.

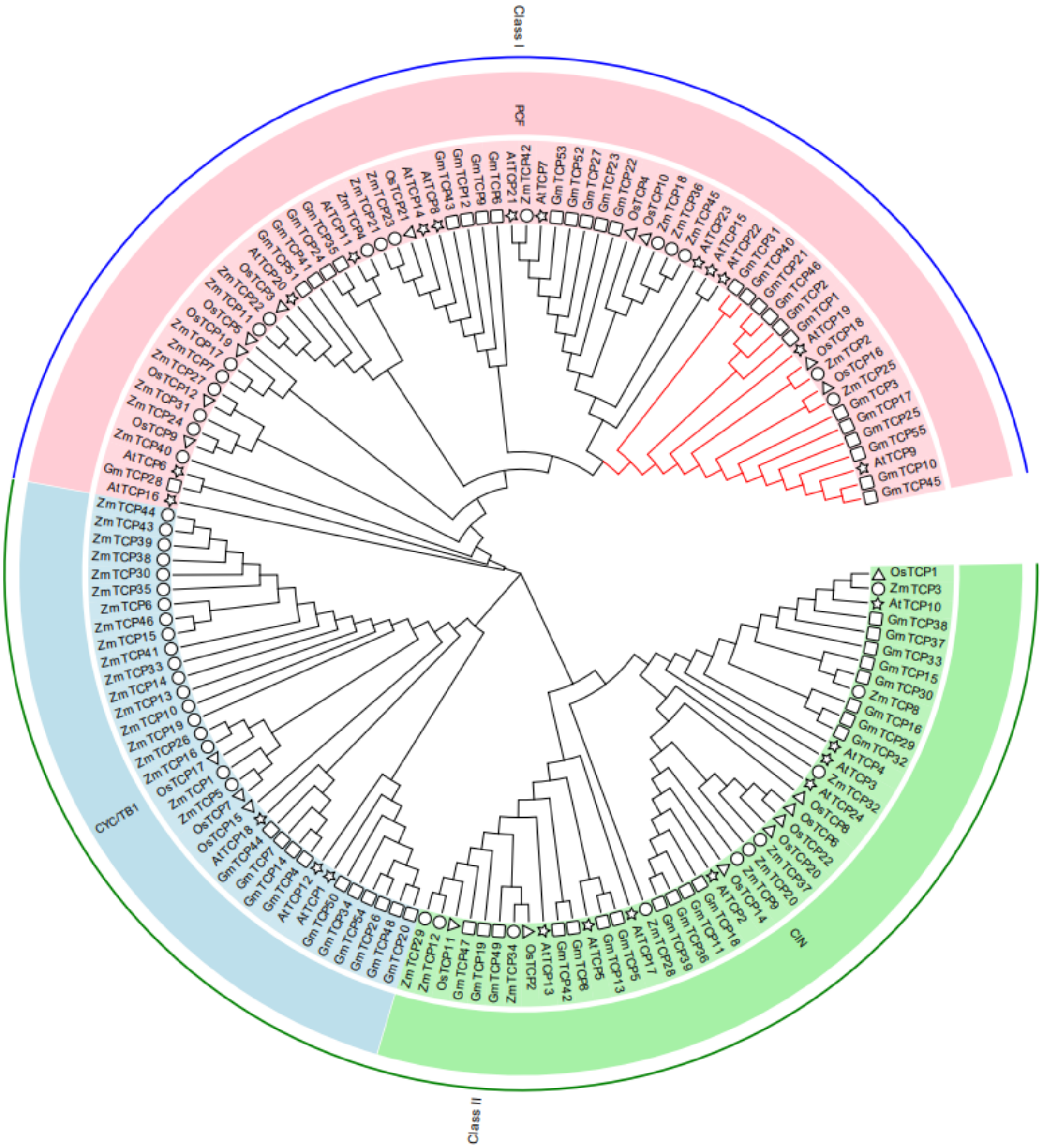

Supplementary Figure S1. Phylogenetic analysis of TCP homologs from four different plant species. Phylogenetic relationships of the TCP proteins from *Arabidopsis thaliana*, *Oryza sativa*, *Zea mays*, and *Glycine max*. The phylogenetic tree was constructed using the neighbor-joining (NJ) method with a bootstrap value of 1000 in MEGA 7 software. The red branch signifies the specific location of GmTCP40 within the branch.

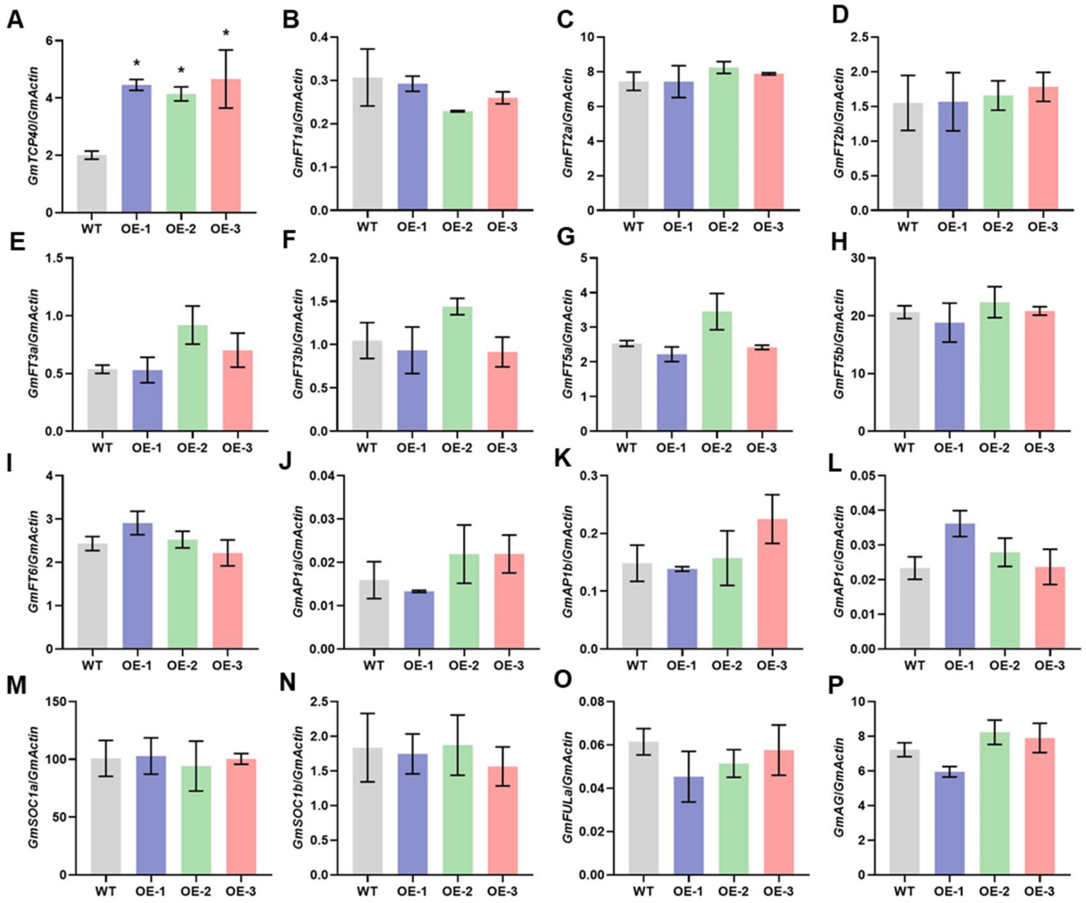

Supplementary Figure S2. Expression levels of flowering-related genes in WT plants and *GmTCP40*-OE transgenic lines under short-day conditions (A-P). The leaves of three *GmTCP40* overexpression lines (OE-1, OE-2, and OE-3) and wild type (WT) plants under short-day (SD) (12 h light/12 h dark) conditions were harvested for qRT-PCR analysis of the expression levels of *GmTCP40* (A), *GmFT1a* (B), *GmFT2a* (C), *GmFT2b* (D), *GmFT3a* (E), *GmFT3b* (F), *GmFT5a* (G), *GmFT5b* (H), *GmFT6* (I), *GmAP1a* (J), *GmAP1b* (K), *GmAP1c* (L), *GmSOC1a* (M), *GmSOC1b* (N), *GmFULa* (O), and *GmAG* (P). The relative expression level was normalized to that of *GmActin*. The data are presented as means ± SD of three replicates (*$p < 0.05$).

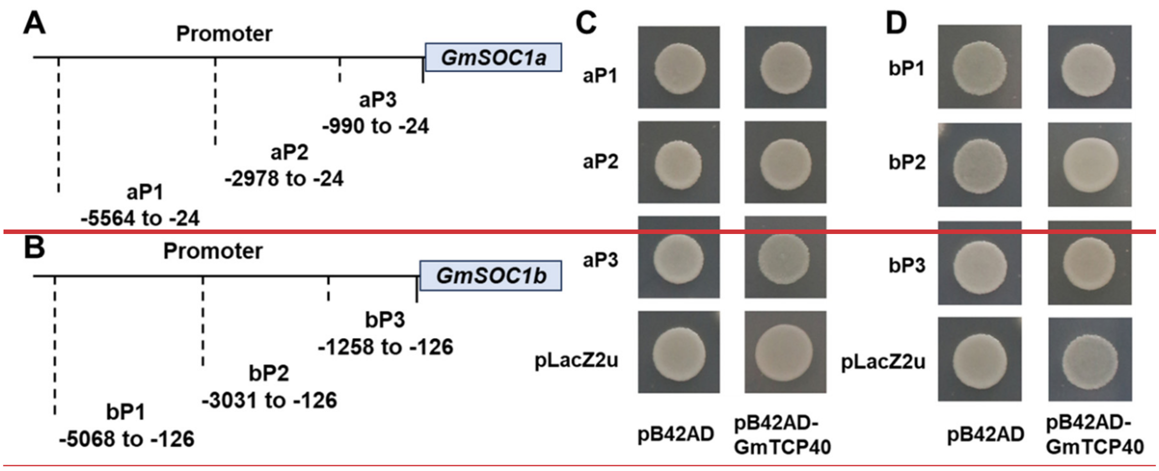

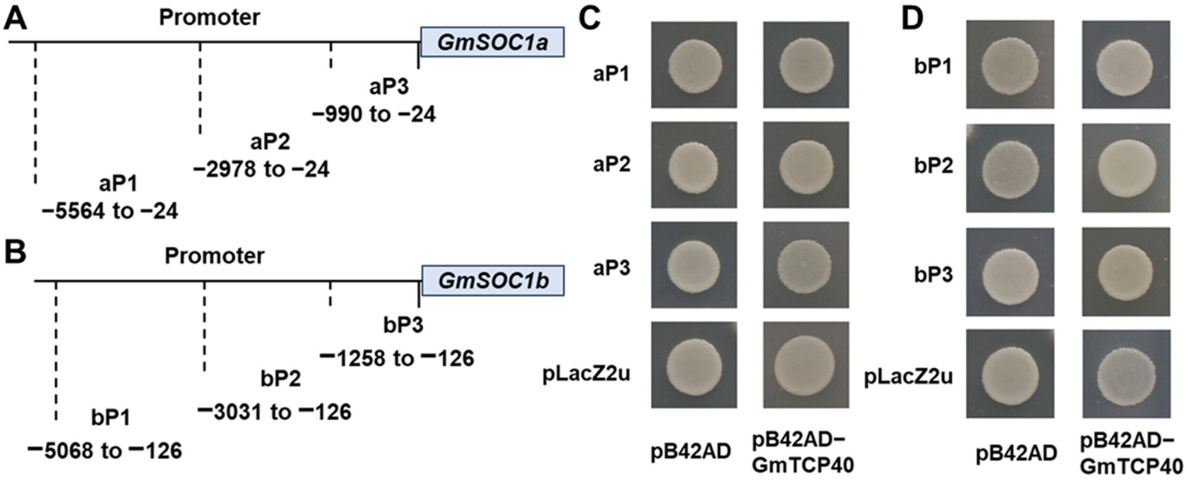

Supplementary Figure S3. GmTCP40 does not bind to promoters of *GmSOC1a* and *GmSOC1b*. (A-B) 3 fragments of the promoter of *GmSOC1a and GmSOC1b* were subcloned into pLacZ2u vector, respectively. (C-D) The interaction between GmTCP40 and the *GmSOC1a and GmSOC1b* promoter was examined by yeast one-hybrid (Y1H) assay. The transformants were assessed on SD/-Trp/-Ura media supplemented with 20 mM X-gal, Galactose (Gal), and Raffinose (Raf). Empty vectors served as the negative controls.

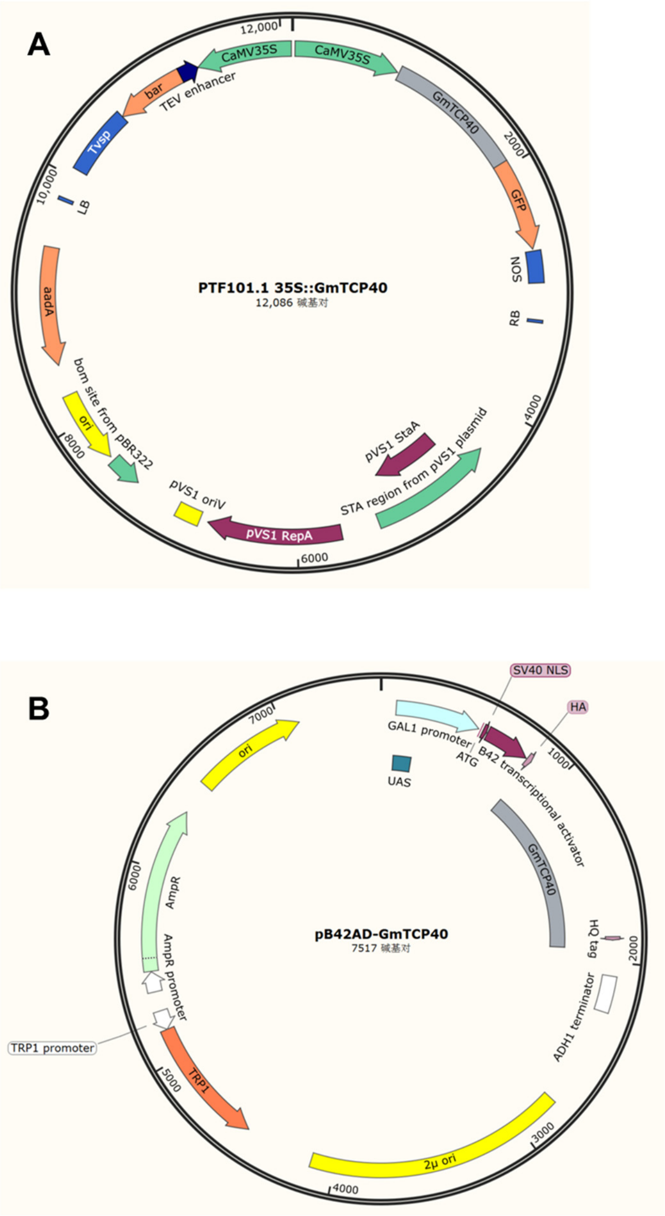

Supplementary Figure S4. Diagrams of pTF101-*GmTCP40* and pB42AD-*GmTCP40* vector. (A) A diagram of the Vector pTF101-*GmTCP40*. (B) A diagram of the Vector pB42AD-*GmTCP40*.

Supplementary Table S1. Source information of 175 re-sequenced soybean varieties.

Variety	Region	Province	Variety	Region	Province
Beihudou	NE	Heilongjiang	Fushou	NE	Inner Mongolia
Beifeng2	NE	Heilongjiang	Jilin4	NE	Jilin
Beifeng9	NE	Heilongjiang	Changnong4	NE	Jilin
Beifeng11	NE	Heilongjiang	Zhi2	NE	Jilin
Beidou5	NE	Heilongjiang	Changnong5	NE	Jilin
Dengke1	NE	Heilongjiang	Fengdihuang	NE	Jilin
Dongnong4	NE	Heilongjiang	Huangbaozhu	NE	Jilin
Dongnong72-806	NE	Heilongjiang	Jilin3	NE	Jilin
Fengshou10	NE	Heilongjiang	Jilin6	NE	Jilin
Suinong14	NE	Heilongjiang	Jilin13	NE	Jilin
Fengshou17	NE	Heilongjiang	Jilin20	NE	Jilin
Fengshou19	NE	Heilongjiang	Jilin30	NE	Jilin
Fengshou24	NE	Heilongjiang	Jilin47	NE	Jilin
Hefeng25	NE	Heilongjiang	Jiti5	NE	Jilin
Hefeng35	NE	Heilongjiang	Jiyu57	NE	Jilin
Zihua4	NE	Heilongjiang	Jiunong9	NE	Jilin
Heihe9	NE	Heilongjiang	Jiunong22	NE	Jilin
Heihe18	NE	Heilongjiang	Xiaojinhuang1	NE	Jilin
Heihe19	NE	Heilongjiang	Zaofeng1	NE	Jilin
Heihe27	NE	Heilongjiang	Jiti1	NE	Liaoning
Heihe38	NE	Heilongjiang	Jin6604-24	NE	Liaoning
Heihe51	NE	Heilongjiang	Kaiyu3	NE	Liaoning
Heihe54	NE	Heilongjiang	Kaiyu8	NE	Liaoning
Heilongjiang41	NE	Heilongjiang	Kaiyu10	NE	Liaoning
Huajiang4	NE	Heilongjiang	Tiefeng3	NE	Liaoning
Jinyuan2	NE	Heilongjiang	Tiefeng8	NE	Liaoning
Jingshanpu	NE	Heilongjiang	Tiefeng18	NE	Liaoning
Kexi283	NE	Heilongjiang	Tiefeng19	NE	Liaoning
Kangxian4	NE	Heilongjiang	Tiefeng20	NE	Liaoning
Mancangjin	NE	Heilongjiang	Dandou2	NE	Liaoning
Suinong3	NE	Heilongjiang	Dandou4	NE	Liaoning
Suinong28	NE	Heilongjiang	Jin8-14	NE	Liaoning
Hejiao6	NE	Heilongjiang	Jin33	NE	Liaoning
Hejiao8	NE	Heilongjiang	Liaodou15	NE	Liaoning
Hefeng22	NE	Heilongjiang	Tiefeng31	NE	Liaoning
Hefeng45	NE	Heilongjiang	Dandou1	NE	Liaoning
Hefeng47	NE	Heilongjiang	Tiefeng29	NE	Liaoning
Hefeng50	NE	Heilongjiang	Changpingqingdou	HHH	Beijing
Hefeng55	NE	Heilongjiang	Jindou2	HHH	Shanxi
Heinong16	NE	Heilongjiang	Jindou3	HHH	Shanxi
Heinong26	NE	Heilongjiang	Jindou19	HHH	Shanxi
Heinong33	NE	Heilongjiang	Jindou21	HHH	Shanxi
Heinong35	NE	Heilongjiang	Jindou25	HHH	Shanxi
Heinong37	NE	Heilongjiang	Jindou1	HHH	Shanxi
Heinong43	NE	Heilongjiang	Hai94	HHH	Shanxi
Heinong48	NE	Heilongjiang	Kefeng6	HHH	Hebei
Kenfeng16	NE	Heilongjiang	Handou5	HHH	Hebei
Suinong10	NE	Heilongjiang	Jidou12	HHH	Hebei
Miquanhuangdou	NE	Xinjiang	Jidou17	HHH	Hebei
Changjihuangdou	NE	Xinjiang	Zhongdou19	HHH	Hebei
Mengdou30	NE	Inner Mongolia	Zhonghuang13	HHH	Hebei

Variety	Region	Province	Variety	Region	Province
Zhonghuang37	HHH	Hebei	Chenliuniumaohuang	SC	Henan
Naiyinheidou	HHH	Hebei	Suidaohuang	SC	Jiangsu
Huairouhuangdou	HHH	Beijing	Nannong493-1	SC	Jiangsu
Qunyingdou	HHH	Hebei	Taixingheidou	SC	Jiangsu
Zhonghuang30	HHH	Hebei	Jinda332	SC	Jiangsu
Zhonghuang35	HHH	Hebei	Edou8	SC	Hubei
Bahong1	HHH	Hebei	Aijiaozao	SC	Hubei
Youbian30	HHH	Hebei	Tianlong1	SC	Hubei
Jidou7	HHH	Hebei	Edou2	SC	Hubei
Hezeniumaohuang	HHH	Shandong	Houzimao	SC	Hubei
Ludou4	HHH	Shandong	Xiangdou3	SC	Hunan
Yanhuang1	HHH	Shandong	Yunyizao	SC	Hunan
Hedou13	HHH	Shandong	Baishudou	SC	Hunan
Fengshouhuang	HHH	Shandong	Qiudou1	SC	Hunan
Yidupingdinghuang	HHH	Shandong	Zigongqingpidou	SC	Sichuan
Wenfeng5	HHH	Shandong	Nandou12	SC	Sichuan
Wenfeng7	HHH	Shandong	Liuyuehuang	SC	Guizhou
Ludou11	HHH	Shandong	Qiandou6	SC	Guizhou
Qihuang10	HHH	Shandong	Shangyukanshanbai	SC	Zhejiang
Haiyangpamanqing	HHH	Shandong	Zhechun3	SC	Zhejiang
Yuejin4	HHH	Shandong	Lanxidaqingdou	SC	Zhejiang
Yuejin5	HHH	Shandong	Jinjiangdaqingren	SC	Fujian
Yudou2	HHH	Henan	Shangraodaqingsi	SC	Jiangxi
Yudou8	HHH	Henan	Fengchengniupidou	SC	Jiangxi
Yudou22	HHH	Henan	Dian86-4	SC	Yunnan
Zheng92116	HHH	Henan	Jinningdahuangdou	SC	Yunnan
Henanzaofeng1	HHH	Henan	Taiwan75	SC	Taiwan
Shangcaiercaoshipingdingshi	HHH	Henan	Pingguohuangdou	SC	Guangxi
Weiqingdou	HHH	Henan	Guichun1	SC	Guangxi
Zhengzhou135	HHH	Henan	Yulindahuangdou	SC	Guangxi
Xudou1	HHH	Jiangsu	Guichun8	SC	Guangxi
Xudou2	HHH	Jiangsu	Baihuadou	SC	Guangdong
Xudou5	HHH	Jiangsu	Juhuangdadou	SC	Guangdong
Xudou9	HHH	Jiangsu	Wuhuasiyuehuang	SC	Guangdong
Wandou24	HHH	Aahui	Yangchunqingpidou	SC	Guangdong
77-14	SC	Jiangsu	Heibiqing	SC	Guangdong
77-158	SC	Jiangsu			

* NE: Northeast of China; HHH: Huang-Huai-Hai; SC: South of China.

Supplementary Table S2. Flowering time of major *GmTCP40* haplotypes across 9 environments with sowing time and day length information

Environments	Sowing time	Day length from sowing to flowering (h)	Flowering time (d)			
			Hap2	Hap3	Hap4	Hap6
SY2016	December 19	14.37	23.78±1.76 a	23.39±1.72 a	23.90±1.57 a	24.34±1.59 a
JN2016	July 3	14.50	27.33±6.32 a	27.70±7.58 a	26.83±4.37 a	34.69±8.99 b
XX2016	July 5	14.44	26.13±6.70 a	25.91±7.54 a	25.34±4.51 a	32.75±9.14 b
BJ2016	July 3	11.89	29.63±7.82 a	30.31±10.14 a	29.17±6.53 a	39.76±11.38 b
XT2016	June 20	14.89	26.47±4.49 a	26.78±5.36 a	26.63±3.18 a	31.00±7.02 b
XX2017	June 22	14.57	25.48±7.88 a	27.11±10.17 a	26.57±6.81 a	37.45±11.71 b
BJ2017	June 17	14.28	29.68±9.68 a	32.91±13.81 a	31.89±9.21 a	47.03±15.22 b
CC2017	May 5	13.11	44.91±16.38 a	50.50±20.08 a	49.62±14.77 a	69.99±19.30 b
HH2017	May 10	16.14	46.99±30.40 a	54.35±29.78 a	64.54±26.61 a	56.55±41.47 b

SY2016, JN2016, XX2016, BJ2016, XT2016, XX2017, BJ2017, CC2017, and HH2017: Sanya (18°18′ N, 112°39′ E) in 2016, Xiangtan (27°40′ N, 112°39′ E) in 2016, Jining (35°26′ N, 116°35′ E) in 2016, Xinxiang (35°08′ N, 113°45′ E) in 2016 and 2017, Beijing (40°13′ N, 116°33′E) in 2016 and 2017, Changchun (43°50′ N, 124°82′ E) in 2017, and Heihe (50°24′ N, 127°49′ E) in 2017, respectively. The data are presented as the means ± SDs, and a and b indicate significant differences determined by Duncan’s test at $p < 0.05$.

Supplementary Table S3 The nucleotide sequences of the primers used in this study.

Primer name	Sequence	Annotation
TCP40-101-F	AGAACACGGGGGACTCTAGAATGAAGAAGCTGACAGGTGG	for cloning GmTCP40
TCP40-101-R	CCCTTGCTCACCATTCTAGATCATTGGGAGCTATTAGGATC	
0800-SOC1a-P1-F	GTCGACGGTATCGATaagcttAGGGTAAGGCTGCGTACAAC	for dual-luciferase assay
0800-SOC1a-P2-F	GTCGACGGTATCGATaagcttGAGGAGAGGGTAATGTCACAAGT	
0800-SOC1a-P3-F	GTCGACGGTATCGATaagcttTCCCAGTGGAAGGGGGTTTA	
0800-SOC1a-P4-F	GTCGACGGTATCGATaagcttACATGACGATCAACGTCCCA	
0800-SOC1a-R	CGCTCTAGAACTAGTggatccTCTCAAAGGAGTCCTAACCAAGT	
62-SK-40-F	GTGGATCCCCCGGGCTGCAGATGAAGAAGCTGACAGGTGG	
62-SK-40-R1	CAGCGTACCGAATTGGTACCTTGGGAGCTATTAGGATCATC	
0800-SOC1b-P1-F	GTCGACGGTATCGATaagcttTTTTTACAGACCTCCTCTAATGAT	
0800-SOC1b-P2-F	GTCGACGGTATCGATaagcttTCCGTTAGAGTCTCACCTTCCT	
0800-SOC1b-P3-F	GTCGACGGTATCGATaagcttTTATGTTGTGGTGGCCTGTA	
0800-SOC1b-R	CGCTCTAGAACTAGTggatccCAAGTAGAATGAACCCAAAA	
0800-AP1a-3K-F	GTCGACGGTATCGATaagcttTGAAACAGAAATTTGGTCAT	
0800-AP1a-3K-R	CGCTCTAGAACTAGTggatccATTACTGTCTTTTTTTTTGC	
SOC1a-P1-p2u-F	CggaattcgagctcggtaccAGGGTAAGGCTGCGTACAAC	for yeast one-hybrid
SOC1a-P2-p2u-F	CggaattcgagctcggtaccGAGGAGAGGGTAATGTCACAAGT	
SOC1a-P3-p2u-F	CggaattcgagctcggtaccTCCCAGTGGAAGGGGGTTTA	
SOC1b-P1-p2u-F	CggaattcgagctcggtaccTTTTTACAGACCTCCTCTAATGAT	
SOC1b-P2-p2u-F	CggaattcgagctcggtaccTCCGTTAGAGTCTCACCTTCCT	
SOC1b-P3-p2u-F	CggaattcgagctcggtaccTTATGTTGTGGTGGCCTGTA	
AP1a-p2u-3K-F	CggaattcgagctcggtaccTGAAACAGAAATTTGGTCAT	
AP1a-p2u-3K-R	TACAGAGCACATGCCTCGAGATTACTGTCTTTTTTTTTGC	
SOC1a-p2u-R	TACAGAGCACATGCCTCGAGTCTCAAAGGAGTCCTAACCAAGT	
SOC1b-p2u-R	TACAGAGCACATGCCTCGAGCAAGTAGAATGAACCCAAAA	
qActin-F142	CGGTGGTTCTATCTTGGCATC	for qPCR
qActin-R142	GTCTTTCGCTTCAATAACCCTA	
TCP40-qPCR-F1	AAGAAGCTGACAGGTGGAGC	
TCP40-qPCR-R1	CAGGCATTCGAATTCTGCGG	
qFT1a-F304	GCCTAGATCAACGGACCCTC	
qFT1a-R304	TCCGAAGTTTGCCCCTGTAG	
qGmFT1b-144-F	TTGAAGTTGGTGGTGATGAC	
qGmFT1b-144-R	CGAAGTTTGCTCCTGTAGTT	
qFT2a-F213	AAGTTGTCAACCAACCAAGGG	
qFT2a-R213	GAATCCCCATCATTGGTCTTGG	
qGmFT3a-F	AGCGCCCAAATAAGAAAGGT	
qGmFT3a-R	TGGTACATATGAGTGCTTCGGT	
qGmFT3b-202-F	CTATGAAAGCCCACGACCC	
qGmFT3b-202-R	TGTTCTACCACCAGAGCCACT	
qFT5a-F139	GATTGGGGATGTTCTCAACCCT	
qFT5a-R139	GTCTTCACCACCAACAGTAACCC	
qGmFT5b-RT-F	GGGTGTGATTGGGGATGTTC	
qGmFT5b-RT-R	CAGTTCCAAGCCATTGCTAAT	
qGmFT6-121-F	CCCTCTTGTTGTTGGACGTG	
qGmFT6-121-R	GGAGGGTTTGAGCTCTCCAC	
qGmAP1a-RT-F	TGAACATGGGTGGCAATTAC	
qGmAP1a-RT-R	TGTCAAATGCCATACCAAAG	
qGmAP1b-RT-F	TGGGAGCAGCCAAACTACAG	
qGmAP1c-RT-F	GAAAGAAAAGGTTGCAGCTTC	
qGmAP1c-RT-R	GCATCCAAGGTGACAGGAAT	
GmSOC1a-RT-F	CGAGTTGCTTTTTTTCCCTAG	
GmSOC1a-RT-R	TGAGTCTTTCCTCTCACCAT	
GmSOC1b-RT-F	AAGAAGCCCAACTGCAATGT	
GmSOC1b-RT-R	GGGCTTCAGAAATGAGGAAAGG	
GmFUL1a-RT-F	CTCCCACAACAACACTAGCTC	

Primer name	Sequence	Annotation
GmFUL1a-RT-R	CCTACAAGACAATTCCAACACGA	
qGmAG-F	CCCACAACAACACTAGCTCTCA	
qGmAG-R	AGTAGTAGCACCCTTCAATT	

Supplementary Table S4 The flowering time of the 175 soybean varieties across different environments.

ID	Flowering time									*GmTCP40* Haplotype
	SY2016	JN2016	XX2016	BJ2016	XT2016	XX2017	BJ2017	CC2017	HH2017	
A001	23.50	22.35	21.70	23.15	23.30	22.00	23.40	40.40	54.80	Hap2
A002	23.05	22.58	22.65	23.45	23.70	20.10	23.60	38.00	51.78	Hap6
A003	22.55	22.05	20.73	22.47	23.60	20.00	23.70	31.67	39.00	Hap3
A004	22.60	20.55	20.69	21.29	23.30	19.80	21.90	28.70	42.70	Hap3
A005	23.30	21.25	19.01	21.54	22.90	20.00	24.30	34.40	58.70	Hap3
A006	22.15	19.60	18.76	21.25	22.00	18.20	20.80	27.60	39.80	Hap2
A007	23.05	21.50	21.05	21.26	23.50	20.50	23.00	29.60	38.40	Hap4
A008	22.10	19.99	17.56	19.17	20.50	16.10	19.50	28.80	35.90	Hap3
A009	23.05	20.40	19.69	21.35	22.50	18.70	22.70	31.50	39.00	Hap3
A010	23.75	22.15	19.52	21.14	23.00	18.00	21.70	30.40	39.60	Hap3
A011	22.85	22.25	23.50	20.75	22.80	19.00	20.90	30.80	40.30	Hap3
A012	23.60	21.10	20.25	20.81	23.20	18.40	19.70	27.10	40.00	Hap4
A013	19.65	21.75	21.30	21.05	21.70	18.40	20.40	31.10	41.90	Hap3
A014	22.80	23.15	22.80	20.85	24.30	19.10	23.10	32.60	61.50	Hap4
A015	22.35	22.90	20.63	24.45	24.40	21.20	25.50	41.30	59.40	Hap3
A016	24.45	23.29	19.90	26.45	24.33	23.30	25.90	47.75	69.70	Hap1
A017	23.70	24.25	22.40	26.39	22.10	22.80	26.50	48.80	69.50	Hap3
A018	21.35	24.20	20.36	23.10	22.30	16.78	22.30	35.80	63.10	Hap3
A019	22.40	25.05	23.05	26.75	25.70	22.17	27.50	44.80	72.20	Hap3
A020	23.00	28.50	25.65	32.71	27.00	29.20	32.90	56.90	78.60	Hap3
A021	22.30	29.50	26.76	32.88	28.30	29.10	37.40	61.00	82.90	Hap3
A022	23.35	24.10	21.23	24.47	23.00	20.70	26.60	40.60	66.70	Hap3
A023	22.00	24.45	22.94	23.86	24.70	21.40	25.50	37.90	62.40	Hap3
A024	24.30	24.95	23.80	28.05	25.20	21.70	27.00	47.63	70.90	Hap3
A025	22.90	29.00	25.19	31.75	28.10	27.40	32.40	54.20	80.20	Hap3
A026	23.50	29.00	27.23	33.55	28.80	31.60	36.70	59.70	89.50	Hap3
A027	22.85	28.50	24.42	33.60	26.00	25.50	36.00	59.60	92.20	Hap3
A028	22.75	27.50	24.95	32.00	25.70	24.40	33.30	58.60	81.80	Hap4
A029	22.85	27.60	25.22	29.35	25.10	27.20	31.00	50.50	72.70	Hap6
A030	23.45	29.50	28.83	34.88	28.20	32.20	38.40	61.90	95.00	Hap3
A031	23.60	28.90	27.46	31.91	25.60	28.80	32.20	56.40	82.90	Hap3
A032	26.45	34.50	31.05	36.76	30.10	37.60	45.40	68.90	Unflowering	Hap6
A033	24.65	32.50	30.63	36.45	30.20	37.90	41.30	64.40	95.43	Hap6
A034	23.95	31.00	31.65	36.05	30.00	31.80	41.70	62.10	92.78	Hap4
A035	25.75	34.00	34.15	38.95	34.80	38.00	44.00	69.80	Unflowering	Hap3
A036	26.30	33.00	32.30	36.33	31.10	33.80	43.10	62.90	86.00	Hap6
A037	26.70	35.70	35.58	41.35	32.70	39.40	46.70	72.40	Unflowering	Hap3
A038	24.20	33.00	30.85	40.48	26.50	36.70	48.10	69.80	96.00	Hap6
A039	24.05	34.00	34.15	41.30	31.90	36.30	44.90	66.60	94.50	Hap3
A040	23.05	32.50	31.05	36.30	24.30	19.60	25.30	38.70	71.90	Hap2
A041	25.55	32.50	32.78	38.45	30.80	36.80	44.70	70.30	Unflowering	Hap4
A042	22.55	33.00	30.10	37.80	28.20	32.60	49.10	77.90	Unflowering	Hap3
A043	23.35	33.00	29.45	37.05	26.50	30.30	47.40	70.60	95.00	Hap3
A044	23.80	31.00	30.15	35.19	29.50	32.50	41.50	64.20	91.40	Hap3
A045	27.35	36.50	36.82	41.85	35.40	39.40	48.00	73.30	Unflowering	Hap3

ID	Flowering time									*GmTCP40* Haplotype
	SY2016	JN2016	XX2016	BJ2016	XT2016	XX2017	BJ2017	CC2017	HH2017	
A046	23.85	44.00	42.80	57.75	36.00	48.40	67.75	96.50	Unflowering	Hap3
A047	29.15	47.00	46.20	54.85	41.90	50.40	66.20	92.80	79.50	Hap3
A048	28.20	40.00	37.15	47.03	36.00	45.60	54.80	80.20	Unflowering	Hap3
A049	26.00	35.50	33.15	41.00	33.90	44.40	47.30	70.40	Unflowering	Hap3
A050	24.75	38.00	35.38	44.55	31.70	42.10	58.00	85.40	Unflowering	Hap6
A051	26.00	40.20	38.40	49.25	32.90	42.90	62.13	91.20	Unflowering	Hap3
A052	25.56	41.00	39.10	50.07	35.00	47.90	63.20	93.00	Unflowering	Hap3
A053	25.60	41.50	37.90	49.43	34.60	45.30	61.90	91.70	Unflowering	Hap3
A054	25.25	39.50	37.92	46.04	34.60	42.80	53.50	84.70	Unflowering	Hap6
A055	26.20	40.00	40.01	49.19	34.60	45.03	63.10	91.40	Unflowering	Hap3
A056	22.55	47.00	43.90	55.76	36.10	50.27	68.15	105.70	Unflowering	Hap6
A057	26.40	61.00	59.70	70.74	50.00	66.30	83.21	109.50	Unflowering	Hap6
A058	25.93	55.00	55.20	67.56	52.40	70.00	82.50	108.90	Unflowering	Hap6
A059	25.62	52.50	50.54	60.69	41.20	60.20	73.79	101.33	Unflowering	Hap6
A060	28.40	46.00	42.20	48.96	44.50	45.50	56.70	86.10	Unflowering	Hap6
A061	22.85	24.05	22.35	23.40	24.40	21.00	24.50	34.80	56.50	Hap3
A062	24.10	29.50	27.11	32.57	27.70	30.98	34.20	56.90	78.00	Hap4
A063	25.00	35.00	34.21	38.69	32.20	36.70	43.90	70.10	Unflowering	Hap2
A064	21.80	31.50	27.50	35.94	26.50	36.60	47.10	72.00	94.00	Hap6
A065	25.60	37.00	36.13	42.10	35.30	39.60	51.00	76.50	Unflowering	Hap6
A066	25.55	35.95	33.84	38.67	31.90	37.00	45.60	65.80	94.00	Hap6
A067	24.25	37.00	37.05	46.81	32.10	42.90	57.40	78.50	Unflowering	Hap6
A068	25.50	69.00	62.05	74.19	56.50	75.20	85.67	114.50	Unflowering	Hap3
A069	23.15	20.85	19.66	22.36	20.90	18.40	20.90	29.60	38.30	Hap3
A070	22.60	19.25	19.11	19.45	21.20	18.50	19.90	29.80	37.00	Hap3
A071	21.10	20.75	18.65	20.90	22.00	18.70	21.10	32.00	45.20	Hap2
A072	21.45	19.75	19.40	19.01	20.00	17.50	19.20	29.20	40.60	Hap3
A073	22.65	20.68	21.25	19.46	21.10	19.50	20.70	30.40	39.80	Hap4
A074	21.80	20.60	18.70	19.68	21.80	17.10	20.30	29.80	38.90	Hap3
A075	22.90	20.69	19.75	21.35	22.60	18.90	21.10	29.20	40.56	Hap3
A076	22.80	19.15	18.75	18.55	20.00	17.40	18.90	27.50	35.30	Hap3
A077	22.00	23.15	21.05	24.51	23.70	20.70	23.30	36.20	60.25	Hap4
A078	22.55	22.30	19.44	23.55	24.20	19.60	22.40	34.50	65.20	Hap3
A079	21.70	22.30	19.75	22.06	24.60	20.10	22.50	36.80	66.60	Hap3
A080	21.05	21.00	21.00	21.98	23.00	19.30	20.80	34.30	45.00	Hap3
A081	23.50	22.95	22.35	23.52	25.70	21.70	23.20	36.20	58.00	Hap4
A082	22.95	22.80	21.17	24.30	25.40	21.70	23.50	36.00	61.40	Hap3
A083	22.25	23.25	20.90	24.92	24.20	21.10	24.00	36.50	58.70	Hap4
A084	22.60	22.95	20.42	22.18	24.60	19.20	22.10	36.60	52.00	Hap4
A085	22.80	23.20	20.80	24.30	24.30	20.40	22.00	31.50	45.30	Hap2
A086	22.65	22.40	21.74	22.90	23.60	21.30	21.50	35.80	55.90	Hap3
A087	23.30	22.93	21.45	23.25	24.70	20.10	22.80	34.90	42.43	Hap4
A088	22.55	33.50	19.44	30.30	24.10	19.60	21.50	30.00	40.83	Hap3
A089	22.60	20.15	18.50	21.05	22.30	19.00	21.50	29.70	42.50	Hap4
A090	22.85	20.95	19.69	22.22	23.40	19.00	22.80	30.40	44.14	Hap3
A091	22.90	22.95	20.30	23.15	23.70	18.90	22.60	32.67	44.10	Hap3
A092	22.70	22.80	20.65	23.00	23.80	20.30	23.80	32.80	53.90	Hap3

ID	Flowering time									*GmTCP40* Haplotype
	SY2016	JN2016	XX2016	BJ2016	XT2016	XX2017	BJ2017	CC2017	HH2017	
A093	22.75	21.60	22.33	23.90	24.10	20.40	23.00	32.10	40.50	Hap3
A094	23.05	21.35	19.45	21.94	22.70	18.60	21.50	29.10	41.43	Hap2
A095	22.00	20.80	20.50	22.70	22.00	20.10	21.60	35.40	54.90	Hap3
A096	23.80	21.80	21.35	21.55	23.30	19.40	23.00	35.30	52.60	Hap3
A097	22.80	22.50	21.90	22.90	23.20	20.80	22.60	36.25	43.13	Hap3
A098	22.10	22.85	21.24	24.67	23.90	19.70	24.00	32.40	43.75	Hap3
A099	22.00	22.60	21.25	23.20	24.40	19.20	23.30	37.90	57.20	Hap3
A100	22.20	22.60	20.88	22.55	24.20	20.30	24.10	33.50	54.10	Hap6
A101	22.15	23.10	19.75	21.94	23.10	19.00	22.50	31.40	46.25	Hap3
A102	22.20	22.50	19.60	20.98	23.80	19.80	23.40	34.00	45.56	Hap3
A103	22.70	22.57	22.50	26.50	23.80	21.00	24.40	38.60	61.90	Hap6
A104	24.00	31.40	28.12	33.71	29.00	33.80	35.60	60.30	83.10	Hap3
A105	24.75	32.50	31.28	36.94	30.70	34.90	40.90	60.70	83.50	Hap3
A106	22.50	28.75	25.20	29.41	24.80	26.90	28.60	46.60	72.80	Hap3
A107	23.45	23.65	20.74	27.10	24.00	21.40	26.50	42.50	72.10	Hap3
A108	23.85	25.15	21.05	29.90	25.60	21.90	28.70	51.70	76.00	Hap3
A109	23.40	26.00	20.70	27.28	24.30	24.00	26.40	47.10	72.50	Hap3
A110	21.40	23.00	21.70	24.79	24.40	20.30	21.00	32.60	63.14	Hap3
A111	21.55	23.60	20.68	22.87	22.50	19.60	22.40	38.30	76.50	Hap3
A112	26.70	25.90	24.75	26.40	30.50	34.60	41.80	65.70	82.33	Hap3
A113	21.45	23.25	22.18	24.83	24.10	20.10	24.10	43.75	67.80	Hap3
A114	22.50	26.80	23.91	28.27	26.30	24.40	29.60	53.10	85.70	Hap3
A115	22.55	24.60	23.54	25.49	24.70	22.80	27.90	47.10	69.67	Hap3
A116	20.90	23.98	20.91	21.84	21.50	18.10	22.20	35.50	59.90	Hap3
A117	22.85	24.67	23.93	27.34	26.10	23.00	26.20	38.10	65.10	Hap3
A118	23.05	25.25	23.60	27.47	25.50	23.40	26.10	39.00	64.90	Hap3
A119	22.85	23.35	21.25	24.08	24.40	20.60	23.70	37.40	67.20	Hap3
A120	21.35	20.90	20.05	22.75	22.40	18.60	21.60	33.40	47.10	Hap3
A121	22.50	29.00	24.85	29.75	26.90	30.20	31.10	53.00	76.40	Hap3
A122	22.60	22.95	20.60	21.52	22.90	20.30	23.40	31.50	67.00	Hap3
A123	24.25	25.15	21.30	24.30	28.70	27.10	32.60	53.70	73.30	Hap3
A124	23.45	29.20	27.30	31.35	28.40	32.20	36.20	56.00	73.60	Hap5
A125	21.75	22.25	20.98	24.74	23.20	21.30	24.40	34.10	66.56	Hap3
A126	22.60	24.95	22.61	26.20	25.30	22.00	24.00	38.78	65.50	Hap3
A127	22.90	24.55	21.48	22.18	24.20	20.30	25.20	41.00	67.10	Hap6
A128	22.05	23.40	21.00	24.25	23.30	20.10	24.70	40.00	68.60	Hap3
A129	22.70	23.75	22.35	24.97	25.60	23.40	26.40	43.60	67.20	Hap4
A130	25.05	30.00	30.05	37.25	25.90	34.40	47.10	66.10	84.10	Hap6
A131	23.15	32.00	33.05	40.24	28.10	37.90	51.40	71.70	90.70	Hap6
A132	23.50	34.00	33.10	40.07	31.30	37.20	44.20	64.60	98.30	Hap3
A133	22.50	31.50	28.45	37.01	26.60	34.80	49.33	71.70	125.00	Hap3
A134	21.25	29.90	29.58	34.87	27.00	27.40	32.80	44.13	67.00	Hap3
A135	23.10	30.00	27.56	37.05	26.10	36.70	46.50	71.10	97.30	Hap6
A136	23.05	26.00	22.05	30.69	23.70	23.30	38.40	58.30	83.10	Hap4
A137	22.15	31.90	24.95	37.50	25.50	35.90	48.80	73.80	99.00	Hap6
A138	21.85	30.00	25.13	35.34	26.30	36.90	47.50	73.10	99.00	Hap4
A139	23.65	32.50	30.00	38.19	30.90	35.20	42.90	63.20	95.60	Hap6

ID	Flowering time									*GmTCP40* Haplotype
	SY2016	JN2016	XX2016	BJ2016	XT2016	XX2017	BJ2017	CC2017	HH2017	
A140	27.60	34.25	33.49	40.12	32.00	34.80	45.20	63.00	98.00	Hap4
A141	23.25	27.75	26.35	31.94	27.40	26.70	34.10	57.20	88.60	Hap6
A142	25.50	34.10	31.80	37.59	33.10	36.40	41.60	65.10	Unflowering	Hap2
A143	25.05	33.45	31.17	37.33	31.70	36.30	44.70	74.80	Unflowering	Hap3
A144	27.70	32.50	30.70	35.84	31.50	32.70	37.00	61.20	86.00	Hap4
A145	25.50	27.40	27.05	34.96	30.70	35.40	41.20	65.70	88.40	Hap4
A146	24.70	28.50	25.59	31.60	28.00	29.20	32.10	57.10	90.30	Hap4
A147	22.15	28.50	27.12	32.30	26.90	28.80	35.70	62.00	83.00	Hap6
A148	23.95	35.50	35.69	41.71	31.80	38.40	47.60	76.50	Unflowering	Hap3
A149	24.05	31.00	31.57	36.80	30.00	33.50	40.00	60.75	83.90	Hap3
A150	22.65	32.00	29.08	35.40	28.50	33.57	38.70	60.80	93.38	Hap3
A151	25.00	28.00	25.73	27.96	24.70	23.00	27.40	47.40	68.30	Hap4
A152	24.35	32.00	33.00	36.83	30.90	36.40	43.20	63.20	94.40	Hap6
A153	23.55	30.00	28.05	34.79	27.00	28.80	35.10	58.20	82.20	Hap3
A154	24.85	32.00	29.90	34.63	30.40	33.40	39.80	60.90	80.50	Hap3
A155	23.65	29.50	26.14	29.35	28.00	26.10	31.30	51.80	71.20	Hap4
A156	26.45	34.00	33.60	39.25	31.90	38.30	43.80	64.78	Unflowering	Hap4
A157	27.10	31.90	32.65	35.27	30.20	30.20	37.50	49.86	77.80	Hap2
A158	24.05	26.30	26.20	27.16	26.70	23.60	28.40	45.30	67.30	Hap4
A159	22.60	23.45	22.75	26.87	23.50	20.30	26.80	44.00	74.80	Hap3
A160	24.75	30.00	29.71	35.45	26.40	35.40	47.40	66.50	85.50	Hap3
A161	22.60	22.70	21.50	25.70	23.30	20.60	24.00	35.00	71.00	Hap3
A162	24.75	32.50	30.90	36.55	32.00	36.60	40.50	71.80	84.00	Hap6
A163	23.55	29.05	25.40	32.15	25.90	29.90	35.10	58.70	82.40	Hap6
A164	27.95	37.50	35.85	40.41	33.40	37.60	47.70	76.50	Unflowering	Hap3
A165	28.05	35.40	37.60	43.14	34.60	39.90	51.40	79.40	Unflowering	Hap3
A166	24.55	32.50	32.20	36.90	30.60	34.00	39.70	64.70	93.70	Hap2
A167	26.05	40.25	37.28	47.20	32.80	43.90	59.40	89.60	Unflowering	Hap6
A168	25.85	26.20	22.55	27.54	24.90	23.20	26.60	45.80	62.00	Hap6
A169	25.60	37.00	35.28	44.00	31.20	40.80	53.90	72.30	97.20	Hap6
A170	23.45	36.50	33.15	41.15	30.30	38.70	50.70	71.10	91.43	Hap6
A171	24.75	32.50	30.65	36.80	30.80	36.80	44.10	69.20	94.83	Hap4
A172	23.60	29.40	28.92	32.70	27.60	30.50	40.80	61.50	82.70	Hap4
A173	26.30	30.00	29.70	33.33	29.90	30.70	36.80	55.10	81.56	Hap3
A174	22.35	23.33	21.16	24.01	23.90	19.10	24.30	41.10	62.60	Hap3
A175	25.50	42.00	44.96	56.43	36.60	48.00	67.17	99.10	Unflowering	Hap3

SY2016, JN2016, XX2016, BJ2016, XT2016, XX2017, BJ2017, CC2017, and HH2017: Sanya (18°18′ N, 112°39′ E) in 2016, Xiangtan (27°40′ N, 112°39′ E) in 2016, Jining (35°26′ N, 116°35′ E) in 2016, Xinxiang (35°08′ N, 113°45′ E) in 2016 and 2017, Beijing (40°13′ N, 116°33′E) in 2016 and 2017, Changchun (43°50′ N, 124°82′ E) in 2017, and Heihe (50°24′ N, 127°49′ E) in 2017, respectively.

Supplementary Table S5 The functional predictions of *cis*-elements identified in the *GmTCP40* promoter.

Element	Motif sequence	Position	Function
TATA-box	ATATAT	239338	core promoter element around -30 of transcription start
TATA-box	TATA	239339	core promoter element around -30 of transcription start
CAAT-box	CAAT	239352	common cis-acting element in promoter and enhancer regions
CAAT-box	CAAT	239379	common cis-acting element in promoter and enhancer regions
TATA-box	ccTATAAAaa	239419	core promoter element around -30 of transcription start
TATA-box	TATAAAA	239420	core promoter element around -30 of transcription start
TATA-box	TATAAA	239421	core promoter element around -30 of transcription start
TATA-box	TATAA	239422	core promoter element around -30 of transcription start
TATA-box	TATACA	239452	core promoter element around -30 of transcription start
TATA-box	TATA	239454	core promoter element around -30 of transcription start
Box 4	ATTAAT	239508	part of a conserved DNA module involved in light responsiveness
CAAT-box	CCCAATTT	239518	common cis-acting element in promoter and enhancer regions
CAAT-box	CCAAT	239519	common cis-acting element in promoter and enhancer regions
CAAT-box	CAAT	239520	common cis-acting element in promoter and enhancer regions
Myb	TAACTG	239556	
TATA-box	ATATAT	239585	core promoter element around -30 of transcription start
TATA-box	TATATA	239586	core promoter element around -30 of transcription start
AT~TATA-box	TATATA	239586	core promoter element around -30 of transcription start
TATA-box	TATA	239588	core promoter element around -30 of transcription start
Box 4	ATTAAT	239607	part of a conserved DNA module involved in light responsiveness
CAAT-box	CAAT	239611	common cis-acting element in promoter and enhancer regions
MBS	CAACTG	239633	MYB binding site involved in drought-inducibility
Myb	CAACTG	239633	
CAAT-box	CAAAT	239639	common cis-acting element in promoter and enhancer regions
ERE	ATTTTAAA	239649	
CAAT-box	CAAT	239655	common cis-acting element in promoter and enhancer regions
TATA-box	ATTATA	239676	core promoter element around -30 of transcription start
TATA-box	TATAA	239677	core promoter element around -30 of transcription start
TATA-box	TATA	239678	core promoter element around -30 of transcription start
TATA-box	TATA	239712	core promoter element around -30 of transcription start
STRE	AGGGG	239724	
CAAT-box	CCAAT	239735	common cis-acting element in promoter and enhancer regions
CAAT-box	CAAT	239740	common cis-acting element in promoter and enhancer regions
WUN-motif	AAATTACT	239754	
TATA-box	TATAAA	239781	core promoter element around -30 of transcription start
TATA-box	TATAA	239782	core promoter element around -30 of transcription start
TATA-box	TATA	239783	core promoter element around -30 of transcription start
CAAT-box	CAAT	239794	common cis-acting element in promoter and enhancer regions
WUN-motif	AAATTTCTT	239825	
GATA-motif	GATAGGG	239861	part of a light responsive element
STRE	AGGGG	239864	
CAAT-box	CAAT	239926	
Box II	TGGTAATAA	239936	part of a light responsive element
AAGAA-motif	GAAAGAA	239973	
Unnamed__4	CTCC	240015	
TATA-box	ccTATAAAaa	240080	core promoter element around -30 of transcription start
STRE	AGGGG	240087	

Element	Motif sequence	Position	Function
MRE	AACCTAA	240121	MYB binding site involved in light responsiveness
Unnamed__4	CTCC	240170	
Unnamed__4	CTCC	240176	
Unnamed__4	CTCC	240179	
Unnamed__4	CTCC	240188	
TATA-box	TATATAA	240195	core promoter element around -30 of transcription start
TATA-box	TATATA	240196	core promoter element around -30 of transcription start
TATA-box	ATATAA	240197	core promoter element around -30 of transcription start
TATA-box	TATA	240198	core promoter element around -30 of transcription start
CAAT-box	TGCCAAC	240207	common cis-acting element in promoter and enhancer regions
TATA-box	ATATAA	240213	core promoter element around -30 of transcription start
TATA-box	TATA	240214	core promoter element around -30 of transcription start
CAAT-box	CCAAT	240221	common cis-acting element in promoter and enhancer regions
TATA-box	TATAA	240315	core promoter element around -30 of transcription start
TATA-box	TATA	240316	core promoter element around -30 of transcription start
TATA-box	TATAA	240361	core promoter element around -30 of transcription start
TATA-box	TATA	240362	core promoter element around -30 of transcription start
Box 4	ATTAAT	240386	part of a conserved DNA module involved in light responsiveness
CAAT-box	CAAT	240415	common cis-acting element in promoter and enhancer regions
CAAT-box	CAAT	240422	common cis-acting element in promoter and enhancer regions
TATA-box	ATTATA	240450	core promoter element around -30 of transcription start
TATA-box	TATAA	240451	core promoter element around -30 of transcription start
TATA-box	TATA	240452	core promoter element around -30 of transcription start
TATA-box	TATA	240495	core promoter element around -30 of transcription start
TATA-box	ATTATA	240501	core promoter element around -30 of transcription start
TATA-box	TATAA	240502	core promoter element around -30 of transcription start
TATA-box	TATA	240503	core promoter element around -30 of transcription start
ERE	ATTTTAAA	240530	
ERE	ATTTTAAA	240532	
TATA-box	ATATAA	240538	core promoter element around -30 of transcription start
TATA-box	TATA	240539	core promoter element around -30 of transcription start
TATA-box	ATTATA	240550	core promoter element around -30 of transcription start
TATA-box	TATAA	240551	core promoter element around -30 of transcription start
TATA-box	TATA	240552	core promoter element around -30 of transcription start
TATA-box	ATATAA	240577	core promoter element around -30 of transcription start
TATA-box	TATA	240578	core promoter element around -30 of transcription start
ERE	ATTTTAAA	240600	
ERE	ATTTTAAA	240602	
ACE	CTAACGTATT	240607	cis-acting element involved in light responsiveness
CAAT-box	CAAT	240636	
TATA-box	ATTATA	240645	core promoter element around -30 of transcription start
TATA-box	TATATAA	240646	core promoter element around -30 of transcription start
TATA-box	TATATA	240647	core promoter element around -30 of transcription start
AT~TATA-box	TATATA	240647	
TATA-box	ATATAA	240648	core promoter element around -30 of transcription start
TATA-box	TATA	240649	core promoter element around -30 of transcription start
CAAT-box	CAAT	240685	
G-box	TAACACGTAG	240700	cis-acting regulatory element involved in light responsiveness

Element	Motif sequence	Position	Function
ABRE	GACACGTGGC	240701	cis-acting element involved in the abscisic acid responsiveness
G-box	ACACGTGGC	240702	cis-acting regulatory element involved in light responsiveness
G-box	CACGTG	240703	cis-acting regulatory element involved in light responsiveness
G-Box	CACGTG	240703	cis-acting regulatory element involved in light responsiveness
ABRE	CACGTG	240703	cis-acting element involved in the abscisic acid responsiveness
ABRE	ACGTG	240704	cis-acting element involved in the abscisic acid responsiveness
Unnamed__1	CGTGG	240705	
CAAT-box	CAAAT	240716	common cis-acting element in promoter and enhancer regions
TC-rich repeats	ATTCTCTAAC	240783	cis-acting element involved in defense and stress responsiveness
MYB-like sequence	TAACCA	240789	
MYB	TAACCA	240789	
CAAT-box	CCAAT	240804	common cis-acting element in promoter and enhancer regions
CAAT-box	CAAT	240826	common cis-acting element in promoter and enhancer regions
TATA-box	ATATAA	240834	core promoter element around -30 of transcription start
TATA-box	TATA	240835	core promoter element around -30 of transcription start
CAAT-box	CAAAT	240839	common cis-acting element in promoter and enhancer regions
TATA-box	ATATAA	240852	core promoter element around -30 of transcription start
TATA-box	TATA	240853	core promoter element around -30 of transcription start
Box 4	ATTAAT	240895	part of a conserved DNA module involved in light responsiveness
TATA-box	TATAAATA	240901	core promoter element around -30 of transcription start
TATA-box	TATAAAT	240902	core promoter element around -30 of transcription start
TATA-box	TATAAA	240903	core promoter element around -30 of transcription start
TATA-box	TATAA	240904	core promoter element around -30 of transcription start
TATA-box	TATA	240905	core promoter element around -30 of transcription start
CAAT-box	CAAT	240924	common cis-acting element in promoter and enhancer regions
CAAT-box	CAAT	240928	common cis-acting element in promoter and enhancer regions
CAAT-box	CAAAT	240954	common cis-acting element in promoter and enhancer regions
CAAT-box	CAAAT	240962	common cis-acting element in promoter and enhancer regions
CAAT-box	CAAT	240993	common cis-acting element in promoter and enhancer regions
TATA-box	ATATAA	241005	core promoter element around -30 of transcription start
TATA-box	TATA	241006	core promoter element around -30 of transcription start
CAAT-box	CAAAT	241016	common cis-acting element in promoter and enhancer regions
Unnamed__4	CTCC	241051	
CAAT-box	CAAAT	241071	common cis-acting element in promoter and enhancer regions
ARE	AAACCA	241074	cis-acting regulatory element essential for the anaerobic induction
TATA-box	TATATA	241099	core promoter element around -30 of transcription start
AT~TATA-box	TATATA	241099	
TATA-box	ATATAT	241100	core promoter element around -30 of transcription start
TATA-box	TATA	241101	core promoter element around -30 of transcription start
GT1-motif	GGTTAA	241110	light responsive element
CAAT-box	CAAT	241135	
L-box	ATCCCACCTAC	241170	part of a light responsive element
TATA-box	ATATAA	241188	core promoter element around -30 of transcription start
TATA-box	TATA	241189	core promoter element around -30 of transcription start
Unnamed__4	CTCC	241229	
CAAT-box	CAAT	241241	common cis-acting element in promoter and enhancer regions
CAAT-box	CAAT	241249	common cis-acting element in promoter and enhancer regions
CAAT-box	CAAT	241273	common cis-acting element in promoter and enhancer regions

Element	Motif sequence	Position	Function
TATA-box	ATTATA	241292	core promoter element around -30 of transcription start
TATA-box	TATATAA	241293	core promoter element around -30 of transcription start
TATA-box	TATATA	241294	core promoter element around -30 of transcription start
AT~TATA-box	TATATA	241294	
TATA-box	ATATAT	241295	core promoter element around -30 of transcription start
TATA-box	TATA	241296	core promoter element around -30 of transcription start
Box 4	ATTAAT	241327	part of a conserved DNA module involved in light responsiveness
GT1-motif	GGTTAA	241398	light responsive element
MYB-like sequence	TAACCA	241399	
MYB	TAACCA	241399	
CAAT-box	CAAT	241411	common cis-acting element in promoter and enhancer regions
TC-rich repeats	GTTTTCTTAC	241419	cis-acting element involved in defense and stress responsiveness
TCT-motif	TCTTAC	241423	part of a light responsive element
MBS	CAACTG	241434	MYB binding site involved in drought-inducibility
Myb	CAACTG	241434	
AuxRR-core	GGTCCAT	241447	cis-acting regulatory element involved in auxin responsiveness
CAAT-box	CCAAT	241452	common cis-acting element in promoter and enhancer regions
CAAT-box	CAAT	241453	common cis-acting element in promoter and enhancer regions
CAAT-box	CAAT	241457	common cis-acting element in promoter and enhancer regions
CAAT-box	CAAT	241481	common cis-acting element in promoter and enhancer regions
CAAT-box	CAAT	241502	common cis-acting element in promoter and enhancer regions
CAAT-box	CAAT	241538	common cis-acting element in promoter and enhancer regions
